# The Association between Gait Speed and Falls in Community Dwelling Older Adults with and without Mild Cognitive Impairment

**DOI:** 10.3390/ijerph18073712

**Published:** 2021-04-02

**Authors:** Claire E. Adam, Annette L. Fitzpatrick, Cindy S. Leary, Anjum Hajat, Elizabeth A. Phelan, Christina Park, Erin O. Semmens

**Affiliations:** 1 School of Public and Community Health Sciences, University of Montana, 32 Campus Drive, Skaggs Building Room 177, Missoula, MT 59812, USA; cindy.leary@umontana.edu (C.S.L.); Erin.Semmens@mso.umt.edu (E.O.S.); 2 Department of Family Medicine, University of Washington, Box #356390, Seattle, WA 98195-6390, USA; fitzpal@uw.edu; 3 Department of Epidemiology, School of Public Health, University of Washington, UW Box #351619, Seattle, WA 98195, USA; anjumh@uw.edu (A.H.); cpark5@uw.edu (C.P.); 4 Department of Global Health, University of Washington, UW Box #351620, Seattle, WA 98195-7965, USA; 5 Division of Gerontology and Geriatric Medicine, Harborview Medical Center, 325 9th Avenue, Box 359755, Seattle, WA 98104-2499, USA; phelane@medicine.washington.edu

**Keywords:** falls, mild cognitive impairment, gait speed

## Abstract

(1) Background: Falls are common in older adults and result in injuries, loss of independence, and death. Slow gait is associated with falls in older adults, but few studies have assessed the association between gait speed and falls among those with mild cognitive impairment (MCI). (2) Methods: The association between gait speed and falls was assessed in 2705 older adults with and without MCI participating in the Ginkgo Evaluation of Memory Study. Gait speed was measured via a 15-foot walk test and fall history through self-report. We used data collected at the 12-month (2001–2003) and 18-month visits (2002–2004). (3) Results: Participant average age was 78.5 years (sd = 3.2); 45% were female, and 14% had MCI at baseline. The average gait speed was 0.93 m/s (sd = 0.20). Sixteen percent (*n* = 433) and 18% (*n* = 498) reported at least one fall at the 12-month and 18-month visits, respectively. Faster gait speed was associated with decreased risk of falling (RR: 0.95, 95% CI: 0.91, 0.99) for every 10 cm/s increase in gait speed adjusted for age, gender, study arm, site, and MCI status. (4) Conclusions: The relationship between gait speed and risk of falling did not vary by MCI status (interaction p-value = 0.78).

## 1. Introduction

Falls in older adults are common, affecting 20 to 33% of those over the age of 65 [1,2]. Death (60 per 100,000 people over 65) [3], injury (46% of falls) [4], medical expenses [3], increased anxiety and depression, decreased quality of life, and loss of independence [5] all result from falls. Given these negative outcomes and that age-adjusted mortality from falls continues to increase [6], screening older adults for fall risk is essential in order for effective fall prevention strategies [7,8,9] to be implemented.

Numerous studies have demonstrated an association between slower gait speed and increased fall risk in the general population of older adults [4,10,11]. However, it is not clear if gait speed is as strongly related to fall risk in those with mild cognitive impairment (MCI) [12], as older adults with MCI have impaired safety awareness and decision making [13] and reduced ability to negotiate obstacles [14]. These impairments are strongly linked to fall risk in a community setting [13,15] but are not tested in well-controlled clinical or research assessments of gait speed, where a participant is asked to walk in a straight line in an environment free of hazards. People with MCI may have decreased executive function, which is a risk factor for falls in older adults, but it is uncertain whether this is adequately tested in gait speed assessments without an added cognitive task [12,13]. To our knowledge, only two studies have examined the relationship between gait speed and falls in a population that included both older adults with and without MCI [16,17]. We hypothesized that gait speed may be more strongly associated with falls in cognitively healthy older adults than those with MCI because factors related to cognition may account for fall risk in older adults with MCI. The objective of this study was to determine if the strength of association between gait speed and falls varied by MCI status in a large population of community-dwelling older adults residing in four, geographically diverse communities in the United States.

## 2. Materials and Methods

Our study population included 3069 adults aged 75 years and older participating in the Gingko Evaluation of Memory Study (GEMS) in four communities in the United States: Sacramento County, CA, Washington County, MD, Forsyth County, NC, and Pittsburgh, PA [18,19]. GEMS was a double-blind randomized controlled trial conducted from 2000 to 2008 designed to investigate if 240 mg/day of *Ginkgo biloba* decreased the risk of Alzheimer’s disease [18,20]. Study methods and their rationale were described in detail in DeKosky et al., 2006 and 2008 [18,20]. Exclusion criteria for participating in GEMS included diagnoses such as Parkinson’s disease, congestive heart failure, recent cancer, and abnormal blood counts [20]. Additionally, older adults taking medication for cognitive function, anti-coagulants, anti-psychotics, and carbidopa/levodopa were excluded from participating in the study [20]. GEMS was a negative study; there were no differences in cognitive outcomes for participants in the placebo vs. intervention group, thus reducing issues with an effect of *Ginkgo biloba* on MCI. GEMS received Institutional Review Board (IRB) approval from all involved sites and this study was additionally approved by the University of Montana IRB.

**Figure 1:**
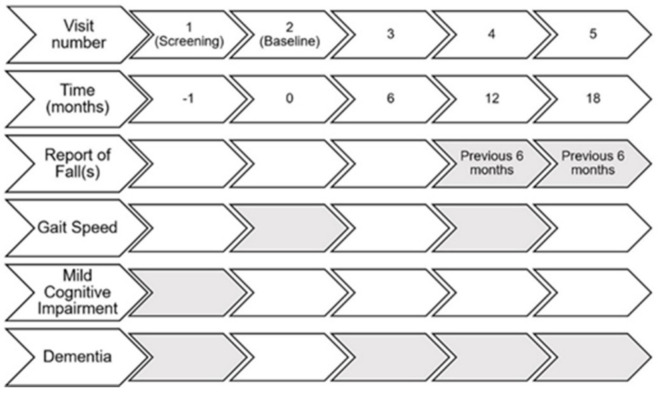
Timeline of GEMS measurements. Shaded boxes indicate when measurement of the variable of interest occurred.

Gait speed in GEMS was assessed annually as part of the Functional Assessment. The gait speed measurements used for this analysis were from the 12-month study visit. The gait speed measurement at the 12-month study visit aligns with the beginning of the 6-month period for falls reported during the fall history at the 18-month visit (Figure 1). Gait speed was measured over a 15-foot walking course with a static start. Participants were initially told to walk 3 feet at their usual pace, and then if able, completed a 15-foot walk test at their usual pace. Participants who had an assistive device for ambulation could use the device during the walk test. Gait speeds faster than 1.79 m/s were excluded for both male and female participants. This is the mean usual gait speed plus 3 standard deviations for men aged 70–79 years over a 4-m walking course with a static start [21]. The gait speed for men aged 70–79 was chosen because it is the fastest gait speed for the age range of male and female participants included in this study [21].

Fall history was ascertained from the Medical History questionnaire completed every 6 months over the course of the study. Participants were asked, “In the past six months since we last saw you, have you had a fall?”, with “yes, no, or don’t know” as possible responses. Any participant who responded “yes” to the question was considered to have had a fall in the past 6 months. Additional information about the definition of a fall was not provided to participants, however at the screening visit, participants were given instructions to not include falls that occurred during skiing, skating, or other activities that may affect balance, but these instructions were not provided at subsequent visits. For this analysis, fall occurrence as a dichotomous variable from the 12-month study visit was assessed as a potential confounder, and fall occurrence as a dichotomous variable from the 18-month study visit was the outcome of interest (Figure 1).

MCI was ascertained at the screening visit and was determined based on criteria from the International Working Group on MCI [22,23]. Study participants who had a score of 0.5 (questionable dementia) on the Clinical Dementia Rating Scale (CDR) and test scores in the 10th percentile or below on at least two out of ten neuropsychological tests, were determined to have MCI [22]. A full description of the methods for determining MCI are available in Snitz et al. 2009 [22]. The prevalence of MCI at the screening visit was 16% [22]. Detailed evaluation of dementia occurred based on the 6-month screening triggered by a participant scoring below threshold on 2 of 3 cognitive tests [modified mini-mental state examination (3MSE), CDR, or Alzheimer’s Disease Assessment Scale-Cognitive Subscale (ADAS-Cog)], new dementia diagnosis by a physician not associated with the study, new memory or cognitive difficulty reported by participant or relative, or starting a medication used to treat cognitive function [19]. Sensitivity analyses were conducted to determine if there was a change in relative risk of falls associated with gait speed if participants who developed dementia by the 18-month study visit were excluded from analyses.

In addition to MCI, treatment arm assignment, and study site; age and gender were selected a priori for inclusion in the modified Poisson regression models for their demonstrated association with falls and gait speed [1,21]. We also considered race, education, 3MSE score, history of cancer, heart attack or stroke, smoking, and alcohol as potential confounders. All data for covariates were collected at the screening or baseline study visit, except for 3MSE score, which was assessed at the 12-month study visit. Information on smoking and alcohol use were obtained from a Health Habits Questionnaire administered at the baseline visit. Smoking status was classified as “never”, “former”, and “current”. Alcohol use was divided into 5 categories based on the number of drinks per week and included “none”, “less than 1”, “1–7”, 7.1–14”, and “more than 14”.

### Statistical Analysis

Participants with data for falls at the 18-month visit, gait speed at the 12-month visit, and MCI, determined at the screening visit, were included in the analysis. Participants were excluded if they were missing data for fall history at the 12-month visit or responded “don’t know” when asked if they had fallen at the 12-month or 18-month visit. Characteristics of excluded and included participants were assessed for statistically significant differences using t-tests and Chi-square tests. We summarized selected characteristics overall and by gait speed quartiles. We used modified Poisson regression with robust standard errors to evaluate associations between gait speed and risk of falling [24]. We chose modified Poisson regression because it does not have the limitations of convergence seen with binomial regression or the overestimation of errors that occurs with ordinary Poisson regression [24]. Covariates in addition to age, gender, treatment, study site and MCI, were included in the model if they altered the relative risk (RR) for gait speed by 10% or greater. We used a staged approach to model building. We assessed effect modification by MCI by including a multiplicative interaction term containing gait speed and MCI in analyses. All analyses were performed with the statistical software R.

## 3. Results

**Figure 2:**
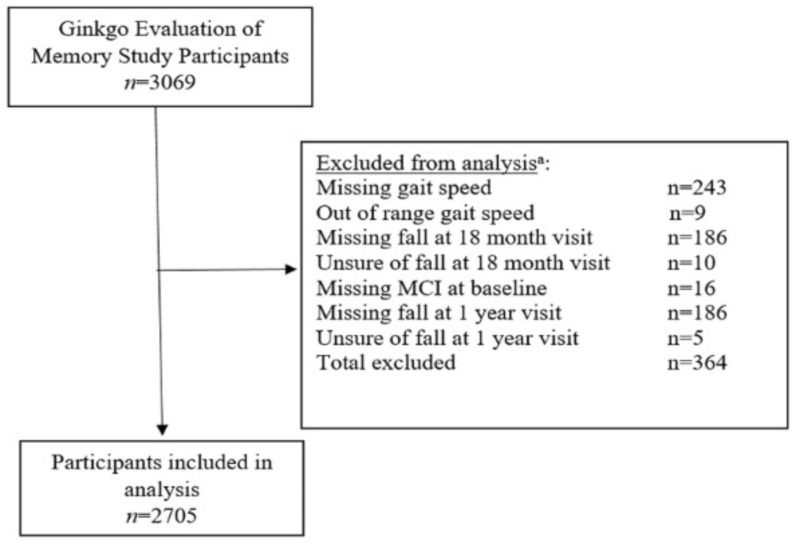
Participants included in analysis. ^a^ Categories are not mutually exclusive; some participants were missing data for multiple measurements.

A total of 2705 study participants were included in the analysis (Figure 2). Of the original 3069 study participants, 364 were excluded from the analysis for missing data, uncertainty about falls, or out of range gait speed (greater than 1.79 m/s) [21] for preferred gait speed (Figure 2).

Within the complete data set of 2705 participants, 45% of participants were female, the mean participant age at baseline was 78.5 (SD = 3.2) years, and 96% of participants were white (Table 1). For health history, 19% had a history of cancer, 10% history of heart attack, 3% history of stroke, and 14% had MCI at the screening visit. The mean 3MSE score at the 12-month visit was 94.4 (SD = 5.0) and 3% used an assistive device at the 12-month visit. For falls, 16% had a fall at the 12-month visit and 18% had a fall at the 18-month visit. The average gait speed at the 12-month visit was 0.93 m/s (0.20). In terms of health habits, 41% of participants never smoked, and 44% of participants did not drink alcohol. Participants excluded from analysis were significantly older (*p* < 0.01), were more likely to be female (*p* < 0.01), were more likely to be from Forsyth County (*p* < 0.01), and were less educated (*p* < 0.01). There were no statistically significant differences in race or treatment arm assignment.

We observed evidence of a relationship between our exposure of interest (gait speed) and age, gender, MCI, study site, a fall reported at the 12-month study visit, education level, 3MSE score, use of an assistive device, history of a stroke, and alcohol use (Table 1).

We observed evidence that our primary outcome of interest (report of a fall at the 18-month visit) was associated with age, study site, fall reported at 12-month study visit, MCI, 3MSE score, use of an assistive device, history of stroke, and history of a heart attack (Table 2).

Following bivariate analysis in addition to the variables specified a priori (age, gender, treatment arm assignment, study site, and MCI), report of a fall at the 12-month study visit was selected as a confounder in the modified Poisson regression models because it changed the relative risk of falls associated with preferred gait speed by 10% [24]. In the unadjusted model including only preferred gait speed at the 12-month visit, a 10 cm/s increase in preferred gait speed was associated with a RR of falling of 0.93 (95% CI: 0.89 to 0.97) (Table 3). In the model adjusted for demographics (age and gender), and treatment arm assignment and study site, a 10 cm/s increase in preferred gait speed was associated with a RR of falling of 0.94 (95% CI: 0.90 to 0.98). In the model adjusted for MCI status, demographics, and treatment arm assignment and study site, a 10 cm/s increase in preferred gait speed was associated with a RR of falling of 0.95 (95% CI: 0.91 to 0.99). In the final model, adjusted for demographics, treatment arm assignment, study site, MCI, and report of a fall at the 12-month study visit, a 10 cm/s increase in preferred gait speed was associated with a RR of falling of 0.96 (95% CI: 0.92 to 1.00). For a model used to assess MCI as an effect modifier, adjusted for demographics, treatment arm assignment, study site, and an interaction term for MCI and gait speed, the p-value for the interaction term was 0.78. For participants without MCI, a 10 cm/s increase in preferred gait speed was associated with a RR of falling of 0.95 (95% CI: 0.90 to 1.00), and for participants with MCI a 10 cm/s increase in preferred gait speed was associated with an RR of falling of 0.94 (95% CI: 0.85 to 1.03). Sensitivity analyses excluding participants diagnosed with dementia by the 18-month visit did not change the relative risk for falls in any of the modified Poisson regression models, except for the RR for people with MCI in the model with the interaction term for MCI and gait speed, however the interaction term remained statistically insignificant (*p*-value = 0.66) (Supplementary Material, Table S1).

## 4. Discussion

We observed a significant association between slower gait speed and increased risk of falling in older adults including those with MCI. This association persisted in models adjusted for age, gender, treatment assignment, study site, and MCI, but not after adjusting for falls at the 12-month visit. Although those with MCI are at a higher risk of falling [17,25], we found no evidence that the association between gait speed and fall risk varied by MCI status. These findings support the use of gait speed as a screening tool for fall risk in both cognitively intact as well as cognitively impaired individuals.

The magnitude of the association between gait speed and falls in our study is similar to those of another study evaluating gait speed as a continuous variable and fall risk [4]. Other studies have found an association between slow gait speed and increased fall risk in a population of people with MCI, but these studies have not specifically examined whether the relationship between gait speed and fall risk is stronger in cognitively intact individuals relative to those with MCI [16,17].

Our study provides evidence that gait speed is a valuable predictor of fall risk even in those with cognitive impairment despite the fact that other factors might influence risk of falls among those with MCI. While these findings are not consistent with our hypothesis, that gait speed may be more strongly associated with falls in cognitively healthy older adults than those with MCI, these findings are consistent with previous work [26]. In a study of older adults with MCI, gait speed and falling were associated, and adding an additional cognitive task to gait speed (dual task) did not improve discrimination between fallers and non-fallers [26]. The identification of potential screening tools for fall risk in older adults with MCI is especially important, given the current lack of recommended screening guidelines for this population [27].

Our study had a number of strengths. Specifically, it benefitted from inclusion of a relatively large and geographically diverse population of older adults for who we have a robust determination of MCI, including multiple diagnostic tests and expert evaluation. In addition, the study included participants who are considered “old (75–84)” and approaching the status of “oldest-old (85 and older)”. These age-groups are at increasingly high risk of falls [1], and screening measures for falls are especially important in this population. There was adjustment for several important underlying chronic conditions associated with falls. We utilized a measure of gait speed that requires minimal space and equipment, making it highly relevant to a clinic setting. Moreover, we found that gait speeds in the study population were consistent with gait speeds observed in adults with this age and gender distribution in previous research [21].

We acknowledge some limitations. While a strength of the study was the inclusion of older adults in the oldest-old age group, the results may not be generalizable to the young-old (65–74 years). In addition, participants were also predominantly white, and the results might not be generalizable to people of other races. Given GEMS exclusion criteria, some chronic conditions were not represented in the data. Missing data were another limitation, with 12% of GEMS participants excluded from the analysis for missing values for falls, gait speed, and MCI. Those excluded from the analysis were more likely to be female, older, and have less education. Fall risk was ascertained by self-report, and was therefore likely underreported in this study; 18% of participants reported a fall; whereas the prevalence of falls found in other studies of adults over 65 is 20% to 33% [1,2]. When examining reported falls by MCI status, 25% of participants with MCI reported a fall, which is within the range reported in the literature for people with MCI [16,28]. However, 17% of participants without MCI reported a fall, which is below the range previously reported for people without cognitive impairment [16,29]. It is possible that underreporting of falls in those without MCI resulted in an underestimate of gait speed associated fall risk in this group. This could have affected our ability to detect a contrast between gait speed impacts on fall risk in those with and without MCI. Finally, while the assessments to determine MCI were robust and the number of participants with MCI was substantial, gait speed and falls were obtained one year and 18 months, respectively, after the initial assessment for MCI. It is possible misclassification of MCI occurred during the follow-up period, as participants may have had a change in MCI status during this time [30]. In a study with a similar population, the Cardiovascular Health Study, over a mean follow-up time of 4.6 years, 18% of people with MCI reverted back to normal cognition, 25% of people with normal cognition developed MCI, and 51% with MCI developed dementia [31]. We were able to address potential misclassification of participants who had MCI or normal cognition at baseline and developed dementia in sensitivity analyses by excluding participants who developed dementia by the 18-month study visit, and results did not change (Supplementary Material, Table S1).

## 5. Conclusions

Our findings add to the evidence that gait speed and fall risk are associated for older adults with and without MCI. Importantly, from our study there was no evidence that the relationship between gait speed and fall risk varied by MCI status, providing support for the use of gait speed as a screening tool for falls for people with and without MCI.

## Figures and Tables

**Table 1:** Participant characteristics by preferred gait speed quartile at 12-month study visit, Ginkgo Evaluation of Memory study (GEMS) (*n* = 2705).

Covariate of Interest	All Participants ^a^ *n* (%)	Gait Speed, Quartile 1 ^b^ *n* (%)	Gait Speed, Quartile 2 ^c^ *n* (%)	Gait Speed Quartile 3 ^d^ *n* (%)	Gait Speed Quartile 4 ^e^ n (%)	*p*-Value (Chi-Square or ANOVA)
Age, years (SD)	78.5 (3.2)	79.5 (3.6)	78.4 (3.2)	78.2 (3.0)	77.8 (2.6)	<0.01
Female	1221 (45)	410 (59)	345 (48)	263 (39)	203 (33)	<0.01
Treatment Ginkgo	1365 (51)	357 (51)	363 (51)	352 (52)	293 (48)	0.60
**Study Site**						<0.01
Forsyth County, NC	623 (23)	181 (26)	180 (25)	148 (22)	114 (19)	
Sacramento County, CA	831 (31)	222 (32)	197 (27)	204 (30)	208 (34)	
Washington County, MD	406 (15)	104 (17)	125 (17)	115 (28)	62 (10)	
Allegheny County, PA	845 (31)	193 (28)	216 (30)	212 (31)	224 (37)	
Fall reported at 12-month visit	433 (16)	149 (21)	110 (15)	95 (14)	79 (13)	<0.01
**Education**						<0.01
High school or less	943 (35)	274 (39)	260 (36)	244 (36)	165 (27)	
Some college	678 (25)	184 (26)	184 (26)	164 (24)	146 (24)	
College graduate	433 (16)	106 (15)	104 (15)	104 (15)	119 (20)	
Postgraduate	651 (24)	136 (19)	170 (24)	167 (25)	178 (29)	
**Health History**						
MCI	383 (14)	139 (20)	94 (13)	78 (12)	72 (12)	<0.01
3MSE score (SD)	94.4 (5.1)	93.1 (5.7)	94.5 (4.8)	94.8 (4.8)	95.3 (4.5)	<0.01
Cancer ^f^	520 (19)	122 (18)	135 (19)	144 (21)	119 (20)	0.35
Heart attack ^g^	255 (10)	69 (10)	69 (10)	62 (9)	55 (9)	0.93
Stroke ^h^	73 (3)	29 (4)	21(3)	13(2)	10 (2)	0.02
**Smoker** ^i^						0.22
Never	1091 (41)	287 (42)	308 (43)	257(38)	239 (40)	
Former	1449 (55)	364(53)	372 (53)	378 (56)	335 (57)	
Current	116 (4)	34 (5)	29 (4)	35 (5)	18 (3)	
**Alcohol Use** **(drinks/week)** ^j^						<0.01
None	1117 (44)	356 (54)	297 (43)	255 (40)	209 (37)	
Less than 1	417 (16)	96 (15)	132 (19)	100 (16)	89 (16)	
1–7	509 (20)	105 (16)	137 (20)	139 (22)	128 (23)	
7.1–14	240 (9)	60 (8)	53 (10)	63 (11)	64 (11)	
More than 14	270 (11)	47 (7)	66 (10)	85 (13)	72 (13)	

Note. Abbreviations: MCI (Mild Cognitive Impairment) ascertained according to 2004 International Working Group criteria, 3MSE (Modified Mini-Mental State Exam) individuals scoring less than 80 at screening were excluded from participating in GEMS [22]. Race and use of an assistive device were excluded from the table, as there were cell counts with fewer than 5 participants; ^a^ *n* = 2705, ^b^ 0.19 m/s to 0.80 m/second ^c^ +0.80 to 0.93 m/s ^d^ +0.93 to 1.06 m/s ^e^ +1.06 to 1.69 m/s; ^f^ History of cancer missing for less than five participants, ^g^ History of heart attack missing for 35 participants, ^h^ History of stroke missing for 49 participants, ^i^ History of smoking missing for 49 participants, ^j^ History of alcohol use missing for 152 participants.

**Table 2:** Participant characteristics by fall status at 18-month study visit, Ginkgo Evaluation of Memory study (GEMS) (*n* = 2705).

Covariate of Interest	All Participants ^a^ *n* (%)	No Fall ^b^ *n* (%)	Fall ^c^ *n* (%)	*p*-Value (Chi-Square or ANOVA)
Age, years (SD)	78.5 (3.2)	78.4 (3.1)	78.9 (3.4)	<0.01
Gender (female)	1221 (45)	982 (44)	239 (48)	0.17
Treatment -Ginkgo	1365 (51)	1116 (51)	249 (50)	0.86
**Study Site**				0.02
Forsyth County, NC	623 (23)	497 (23)	126 (25)	
Sacramento County, CA	831 (31)	668 (30)	163 (33)	
Washington County, MD	406 (15)	323 (15)	83 (17)	
Allegheny County, PA	845 (31)	719 (33)	126 (25)	
Fall reported at 12 month visit	433 (16)	274 (12)	159 (32)	<0.01
**Education**				0.34
Highschool or less	943 (35)	783 (36)	160 (32)	
Some college	678 (25)	545 (25)	133 (27)	
College graduate	433 (16)	358 (16)	75 (15)	
Postgraduate	651 (24)	521 (24)	130 (26)	
**Health History**				
MCI (yes)	383 (14)	289 (13)	94 (19)	<0.01
3MSE score (SD)	94.4 (5.1)	94.5 (5.0)	93.9 (5.3)	0.02
Use assistive device	86 (3)	52 (2)	34 (7)	<0.01
Cancer ^d^	520 (19)	424 (19)	96 (19)	0.98
Stroke ^e^	73 (3)	52 (2)	21 (4)	0.02
Heart attack ^f^	255 (10)	193 (9)	62 (13)	0.01
**Smoker** ^g^				0.16
Never	1091 (41)	877 (40)	214 (44)	
Former	1449 (55)	1202 (55)	247 (51)	
Current	116 (4)	91 (4)	25 (5)	
**Alcohol Use** **(drinks/week)** ^h^				0.57
None	1117 (44)	899 (43)	218 (47)	
Less than 1	417 (16)	347 (17)	70 (15)	
1–7	509 (20)	424 (20)	85 (18)	
7.1–14	240 (9)	193 (9)	47 (10)	
More than 14	270 (11)	222 (11)	48 (10)	

Note. Abbreviations: MCI (Mild Cognitive Impairment), 3MSE (Modified Mini-Mental State Exam), Hx (history). Race was excluded from the table, as there were cell counts with fewer than 5 participants. ^a^ *n* = 2705 ^b^ *n* = 2207 ^c^ *n* = 498; ^d^ History of cancer missing for less than five participants, ^e^ History of heart attack missing for 35 participants, ^f^ History of stroke missing for 49 participants, ^g^ History of smoking missing for 49 participants, ^h^ History of alcohol use missing for 152 participants.

**Table 3:** The association (RR, 95% CI) between preferred gait speed (10 cm/s) and falls in 2705 older adults participating in GEMS.

Model	Relative Risk	95% CI
Unadjusted model	0.93	0.89 to 0.97
Model adjusted for age, gender, treatment arm, and study site	0.94	0.90 to 0.98
Additional adjustment for MCI	0.95	0.91 to 0.99
Additional adjustment for fall at 12-month visit	0.96	0.92 to 1.00
With MCI, additional adjustment for interaction between MCI and gait speed *	0.94	0.85 to 1.03
Without MCI, additional adjustment for interaction between MCI and gait speed *	0.95	0.90 to 1.00

* *p* = 0.78 for interaction between gait speed and MCI.

## Data Availability

Data are not publicly available and data sharing is not applicable to this article.

## Supplementary Materials

The following are available online at https://www.mdpi.com/article/10.3390/ijerph18073712/s1. Table S1: Sensitivity analysis, the association (RR, 95% CI) between preferred gait speed (10 cm/second) and falls in 2679 older adults participating in GEMS, excluding participants diagnosed with dementia by the 18-month study visit.
