# Supplementary material for: The Association between Gait Speed and Falls in Community Dwelling Older Adults with and without Mild Cognitive Impairment
Source: Int J Environ Res Public Health. 2021 Apr 2;18(7):3712. doi: 10.3390/ijerph18073712 (PMC8038190; doi:10.3390/ijerph18073712)
Supplement: Supplementary file 1 [file ijerph-18-03712-s001.pdf]

Supplementary Material.

Table 1. Sensitivity analysis, the association (RR, 95% CI) between preferred gait speed (10 cm/second) and falls in 2,679 older adults participating in GEMS, excluding participants diagnosed with dementia by the 18-month study visit.

Model	Relative Risk	95% CI
Unadjusted model	0.93	0.89 to 0.97
Model adjusted for age, gender, treatment, and clinic	0.94	0.90 to 0.99
Additional adjustment for MCI	0.95	0.90 to 0.99
Additional adjustment for fall at 12-month visit	0.96	0.92 to 1.00
With MCI, additional adjustment for interaction between MCI and gait speed*	0.93	0.85 to 1.02
Without MCI , additional adjustment for interaction between MCI and gait speed*	0.95	0.90 to 1.00

*p=0.66 for interaction between gait speed and MCI.
